# Co-design of the Australian Prompt Response Network for a public-health focused intersectoral approach to information sharing on emerging drugs of concern

**DOI:** 10.3389/fpubh.2025.1521911

**Published:** 2025-06-25

**Authors:** Krista J. Siefried, Penny Hill, Brendan Clifford, Jared Brown, Andrew Camilleri, Sione Crawford, Paul Dessauer, Ella Dilkes-Frayne, Jack Freestone, John Gobeil, Mary E. Harrod, Suzie Hudson, Philip Hull, Simon Lenton, Tom Lyons, Grace Oh, Amy Peacock, Alice Pierce, Peter Sidaway, Jessamine Soderstrom, Stephanie Tzanetis, Nadine Ezard

**Affiliations:** ^1^The National Centre for Clinical Research on Emerging Drugs, The University of New South Wales, Sydney, NSW, Australia; ^2^St Vincent's Hospital Alcohol and Drug Service, Darlinghurst, NSW, Australia; ^3^The National Drug and Alcohol Research Centre, the University of New South Wales, Randwick, NSW, Australia; ^4^Drug and Alcohol Clinical Research and Improvement Network, Sydney, NSW, Australia; ^5^Centre for Alcohol and Other Drugs, New South Wales Ministry of Health, St Leonards, NSW, Australia; ^6^New South Wales Poisons Information Centre, Sydney Children's Hospital Network, Westmead, NSW, Australia; ^7^Forensic Science SA, Adelaide, SA, Australia; ^8^Harm Reduction Victoria, Melbourne, VIC, Australia; ^9^Peer Based Harm Reduction WA, Perth, WA, Australia; ^10^Population Health Division, ACT Health Directorate, Canberra, ACT, Australia; ^11^Australian Injecting and Illicit Drug Users League (AIVL), Sydney, NSW, Australia; ^12^The NSW Users and AIDS Association (NUAA), Sydney, NSW, Australia; ^13^National Drug Research Institute and enAble Institute, Curtin University, Perth, WA, Australia; ^14^Victorian Department of Health, Melbourne, VIC, Australia; ^15^Australian Drug Education & Consultancy, Bellevue, WA, Australia; ^16^School of Psychological Sciences, University of Tasmania, Hobart, TAS, Australia; ^17^Northern Territory AIDS and Hepatitis Council (NTAHC), Darwin, NT, Australia; ^18^Emergency Medicine, Royal Perth Hospital, Perth, WA, Australia; ^19^Centre for Clinical Research in Emergency Medicine, Harry Perkins Institute of Medical Research, Perth, WA, Australia; ^20^Pill Testing Australia, Canberra, ACT, Australia; ^21^Directions Health Services, Canberra, ACT, Australia; ^22^Canberra Alliance for Harm Minimisation & Advocacy (CAHMA), Canberra, ACT, Australia

**Keywords:** co-design, co-creation, emerging drugs, drug risk communication, early warning system, new psychoactive substances (NPS), counterfeit pharmaceuticals

## Abstract

The rapid emergence of new psychoactive substances (NPS) and other emerging drugs of concern presents a significant global public health challenge, necessitating agile and interconnected drug information systems to identify and communicate risks. In Australia, responses have traditionally been localized, lacking a nationally coordinated system to rapidly share information about emerging drug threats. The National Center for Clinical Research on Emerging Drugs (NCCRED) collaborated with jurisdictional networks, clinicians, scientists, policy-makers, and peer organizations to co-design and co-produce the national Prompt Response Network (“PRN”). This process identified key components necessary to create an effective health-focused national network that supports and enhances existing and emerging jurisdictional and specialist early warning networks. The co-creation process resulted in several outputs, including a formalized national PRN group, an online knowledge exchange platform, a national website for disseminating drug alerts, and identified needs for a national drug signal database and an anecdotal reporting system. The PRN is the first Australian national public-health-focused mechanism for information exchange on new and emerging drugs and drug trends of concern. It provides the means for timely and responsive sharing of localized data, better informing risk assessment and facilitating a coordinated approach to public health responses and local and national preparation for emerging risks. Achieving this required mobilizing diverse disciplinary and community stakeholders toward a unified and collaborative response to preventing drug related harms.

## Introduction

### Background

Changes in drug markets and the rapid emergence of new psychoactive substances (NPS) are a key public health challenge in Australia and internationally (1, 2). NPS encompass a variety of emerging drugs of concern, including synthetic cannabinoids, new psychostimulants and hallucinogens, and synthetic opioids (such as fentanyl analogs and nitazenes) (3). As of November 2023, 1,230 NPS were reported to the United Nations Office on Drugs and Crime (UNODC) Early Warning Advisory (4). People use NPS seeking new experiences, altered states of consciousness, or to circumvent legal restrictions on traditional drugs (5, 6). In addition to NPS, broader shifts are evident in drug markets. For instance, there are increasing reports of traditional illicit substances adulterated with unexpected substances (e.g., stimulants containing opioids such as nitazenes) (7), a rise in counterfeit pharmaceutical products (8), and high dose formulations of known substances with unpredictable effects (9). These changes can result in severe or unique clinical presentations or death. Additionally, emerging harms associated with known drugs [e.g., gamma hydroxybutyrate (10)] as prevalence or contexts of use evolve can increase public health risks. This dynamic landscape has challenged existing approaches and demands more rapid identification, communication and response to risks.

Effective public health responses to drug-related risks require adaptive and coordinated approaches, bringing together expertise from a range of stakeholders across clinical, toxicological, forensic, emergency departments, health regulators, public health, alcohol and other drug sector, law enforcement, customs, community sectors, researchers, and importantly, people with lived and living experience of drug use. Without such coordination, delays in detecting and responding to health problems associated with emerging drugs of concern can hinder reliable information sharing and appropriate health interventions. All stakeholders must share a common focus on preventing and minimizing drug-related harms, rather than pursuing other agendas, such as prosecuting drug laws. Internationally, monitoring systems have had to evolve in response to these circumstances, enhancing their capacity to monitor and respond to trends in drug use and availability, and to communicate data on these trends to key stakeholders (11, 12).

### Rationale for innovation

Recent clusters of drug toxicity-related harms in Australia have emerged across various settings and substances, such as high dose MDMA at music festivals (13), nitazenes adulterating cocaine at party and nightclub venues (7), and high levels of thebaine in poppy seed tea in private dwellings (14). Historically, delays in detection and response within public-health frameworks focused on harm reduction have contributed to stigmatizing and alarmist media responses, as was seen with methamphetamine between 2014 and 2016 (15). Additionally, historical lack of information sharing between fragmented services (e.g., jurisdictional health services, local health districts, non-governmental, and peer organizations) has resulted in disparate approaches to health information sharing, education, and resource development (16).

Australia's federation comprises six states and two more populous territories [the Australian Capital Territory (ACT) and Northern Territory (NT)], with health services typically operated at the state/territory level. State-based examples of early warning systems have been successful. Federated governance is a common limitation to national coordination in the Australian context. However, recent coronial inquests (13, 17) and parliamentary inquiries (18, 19), have highlighted the need for a nationally coordinated approach to information sharing.

As part of the National Ice Action Strategy (20) and in response to growing concern about methamphetamine and other emerging drugs, the National Center for Clinical Research on Emerging Drugs (NCCRED), a practitioner academic group, was formed. Funded by the Australian Commonwealth Department of Health and Aged Care, NCCRED is a consortium comprising the National Drug and Alcohol Research Center (University of New South Wales), the National Drug Research Institute (Curtin University), the National Center for Education and Training on Addiction (Flinders University), and St Vincent's Health Australia.

A key goal of NCCRED is to facilitate the co-design and co-creation of a national approach to establish a transparent and responsive information-sharing network for emerging drugs of concern. This involves understanding the roles and activities of jurisdictional (i.e., state and territory) health departments, peer and consumer-focused non-governmental organizations, toxicological and academic groups, and law enforcement. This paper describes the co-creation of the national Prompt Response Network (PRN), which aims to integrate existing and emerging networks and related stakeholders across Australia to share information and knowledge, facilitating timely public health responses to reduce harms from emerging drugs of concern.

## Context

Drug information systems have traditionally focused on routine monitoring, such as coronial reporting (21), law enforcement/border control drug seizures (22), and wastewater analysis (23). However, there are often significant delays in the sharing of these data and reporting timeframes. While these systems can identify trends over time, they do not facilitate early signal detection. With the advent of NPS and other emerging drugs, systems have been implemented to identify high-risk threat signals earlier.

In Australia, formal signals for emerging drugs of concern are detected through methods such as emergency department toxicosurveillance (24–27), quantitative analysis of substances obtained at venues like music festivals (28), and syringe residue testing (29, 30). Government-sanctioned/supported/funded drug checking has been limited but is developing. A fixed-site testing service was established in the Australian Capital Territory (reaching about 1.7% of Australians) (31) in 2018, and while a service recently commenced in Queensland (32), it has since been ceased. A limited service has commenced at the Medically Supervised Injecting Center in Kings Cross, Sydney, and New South Wales trialed a drug checking service at festivals in the 2024–25 season, while a comprehensive drug checking service has recently been announced in Victoria (33). Other Australian states and territories are observing these developments, but drug checking services are not yet available in other Australian jurisdictions.

Anecdotal information is shared among people who use drugs, and when appropriate, consumer organizations (i.e., peer-based, not-for-profit, and/or non-governmental) will triage information and determine when to communicate anecdotal risk externally.

In instances when more current data are available, such as hospital or emergency services presentations, each jurisdiction (e.g., state or territory health departments) assesses the risk to determine when to deliver health information to frontline clinicians or issue a public health alert about harmful effects. In some jurisdictions, clinicians and consumer organizations are involved in the risk assessment process. In disseminating such alerts, peer and consumer-led non-governmental organizations (34) are crucial in ensuring the information reaches people who use drugs, as are informal peer-to-peer networks (35).

NCCRED proposed implementing the PRN in partnership with stakeholders across the sector. The mission was to coordinate participants to share information and knowledge for timely public health responses, reducing the harmful effects of emerging drugs. Crucially, the knowledge of people who use drugs is central to ensuring effective and equitable systems (36). This paper describes the methods undertaken through collaborative consultation to co-design and co-create the national Prompt Response Network.

## Process

### Co-creation

Monitoring for and responding to emerging drugs of concern necessarily involves a broad group of stakeholders, including people who use drugs, policymakers, researchers, clinical and forensic toxicologists, treatment services, emergency services, clinicians, and harm reduction providers. Co-creation is based on effectively engaging diverse stakeholders to understand complex issues and create services and products of value (37). It extends from co-design to co-production, resting on guiding principles including equal partnership, inclusivity of all perspectives and skills, reciprocity and building and maintaining of relationships. A co-creation process was commenced in line with best practice (38) and following a model (39) that included steps to identify, analyse, define, design, and realize the program of work (38, 39).

#### Identify and analyse

The initial step of co-creation involves identifying relevant systems and stakeholders (39). Initial meetings were conducted by an expert in drug policy (author SH) with between eight and 15 relevant stakeholders in each state and territory, followed by 17 individual sessions with additional stakeholders identified through preliminary meetings. This process included jurisdictional representatives from state, territory and federal Government agencies; clinicians, toxicologists, peer organizations, people with lived and living experience and consumer organizations that represent them. Consultations spanned prior to and during COVID-19 lockdowns, benefitting from pivots to online videoconferencing. Reimbursement to peer organizations (e.g., monthly for 2 h' time) was essential in enabling engagement by providing justification for resources.

Through these consultations, a wide range of existing systems of relevance to a PRN were identified, such as sentinel drug monitoring in emergency department presentations (25), regular surveys of people who use drugs (40), and routine analyses of (lagged) administrative data. Differences in jurisdictions were also identified, such as NSW having developed systems with analysis of police seizures with a public health focus in 2019. Through consultation, an understanding was developed of what information can and should be shared and how. *Ad-hoc* information sharing, informal processes, and limited communication between jurisdictions were identified. Amongst stakeholders, limited resourcing was common, and much existing monitoring and communication work relied on good will and informal interpersonal relationships to build trust and engagement, particularly given the potential (described above) for media sensationalism, and the wariness between stakeholders from various sectors such as law enforcement and people with living experience of drug use.

The value of localized expertise and building trust and relationships was pivotal to shared goals with key groups, including police, paramedics, and health professionals when sharing information, and particularly when responding to media. The key issues identified for a national network to support prompt responses to emerging drugs included the need for closed networks for secure information sharing, the importance of proper data governance, and how effective risk communication relied on local knowledge. It was emphasized that national efforts should not aim to impose a top-down solution that would be unfeasible in local contexts and needed to value local experience, expertise, and contextual factors. A shared recognition of the necessity of the work was evident, and a commitment and willingness to enable a national network was established.

#### Define

A national workshop was convened in September 2020 with all participants to synthesize the findings of the consultations and outline priorities for further co-design activities. Key principles identified for further work included establishing a network approach to facilitate knowledge exchange and information dissemination necessary for timely health responses and decision-making. A facilitated network to provide opportunities for shared information through traditional means (online meetings), and innovative means (online platforms) was clarified as the preferred initial approach.

Stakeholders also highlighted the need to co-design digital platforms for confidential information sharing at early or undefined stages of emerging drug risks and an outward facing information sharing platform. For some jurisdictions, these platforms needed to integrate with existing early warning information systems, while for others, there was no such system in place at that stage.

There was also consensus to formalize the group of PRN stakeholders created during the consultation phase into a formal national network facilitated by NCCRED.

#### Design

Following national consultations, from 2021 through 2023, the design phase focused on translating the identified priorities into actionable steps. This phase emphasized the collaborative development of digital platforms for confidential information sharing and public dissemination, and was led by a co-design team of author PH (a research fellow at NCCRED) and an external consultation agency specializing in collaborative, user-centered design [Collabforge (41)]. The involvement of an external consultant supported open dialogue and engagement across diverse groups where perspectives or priorities might differ.

To facilitate this, two reference groups were formed, each focusing on specific aspects of the network. The Jurisdictional Representative Group comprised people involved in key decisions in the public sector within each state and territory. The Peer Advisory Group had representatives from peak organizations representing people who use drugs in each state and territory. The reference groups provided oversight and guidance on technical infrastructure, digital products, data governance, communication strategies, sense-checked the alignment with local processes, and started the initial national knowledge exchange between jurisdictions. The reference groups fed into quarterly national PRN meetings where other relevant stakeholders could discuss new and emerging drugs of concern and share updates on the development of early warning systems in their own jurisdictions.

**Figure 1:**
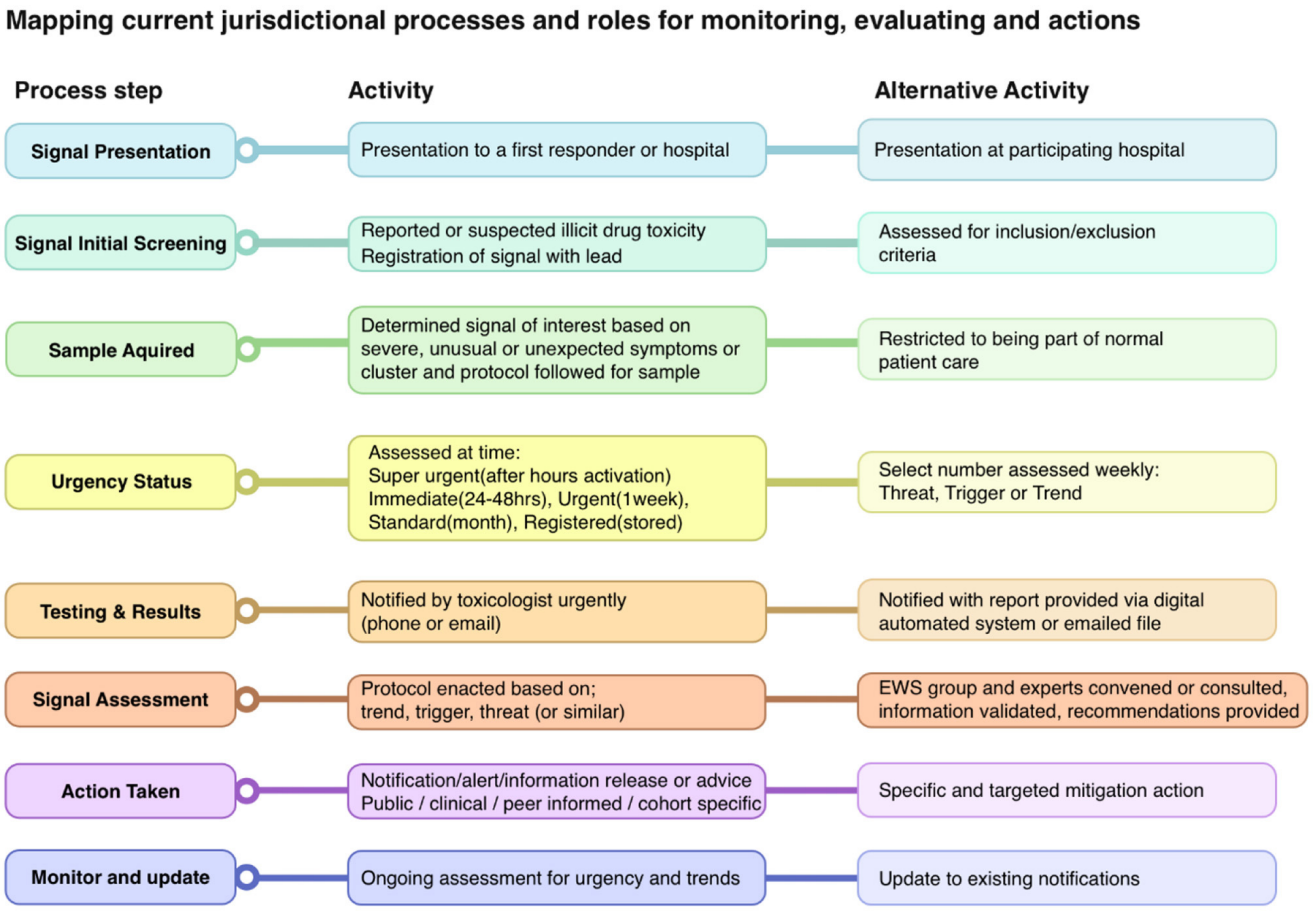
Stakeholder mapping.

Having identified the need for digital platforms to support the activity of the PRN, further process mapping was required to clarify how these products should engage with existing systems within jurisdictions. Process mapping allows for the creation of a shared understanding of complex systems, identification of gaps and improvement opportunities, and provides a starting point to identify shared project objectives (42). Key stakeholders from each jurisdiction were invited to online meetings with the co-design team. They mapped their existing activities, processes, and roles in monitoring and evaluation for identifying drugs/signals as they emerged in their jurisdiction. This included documenting existing steps and processes, noting which experts/organizations were involved and when, key decision-making points, and what networks and communications were utilized. These were mapped (Figure 1), resulting in an extensive shared understanding of processes.

Ongoing dialogue and reflection were conducted with participants throughout the process. Further workshops with the co-design team, information technology (IT) consultants (systems architects), and stakeholders defined the shared understanding of the purpose and goals of the initial systems. Interviews with collaborating stakeholders from four jurisdictions were conducted to understand the technology currently being used for signal monitoring and communication, and possible requirements for integrating local systems with a national network were identified. The IT consultants attended three demonstrations with relevant technology vendors to determine which off-the-shelf software might be cost effective and suitable for stakeholder's needs. These were then presented to the co-design team with a range of recommendations for technology, based on the relationships between, contributors to, and access pathways available through the proposed network.

Two key elements were suggested: (i) an online community management system to facilitate networking, communication, and information sharing throughout the national PRN; and (ii) a searchable national database that could synthesize local signal and alert data, with appropriate data cleaning, storage, searching, and incident and alert management.

#### Realize

Drawing from the insights and direction provided through the co-design process, the PRN was co-produced consisting of the following key pillars: (1) a formalized national PRN group who engage in information sharing; (2) an online knowledge exchange platform (“The Know Community”); (3) a public website (http://theknow.org.au); and components still in development; (4) the national database for drug signals, and (5) an anecdotal reporting system.

**Figure 2:**
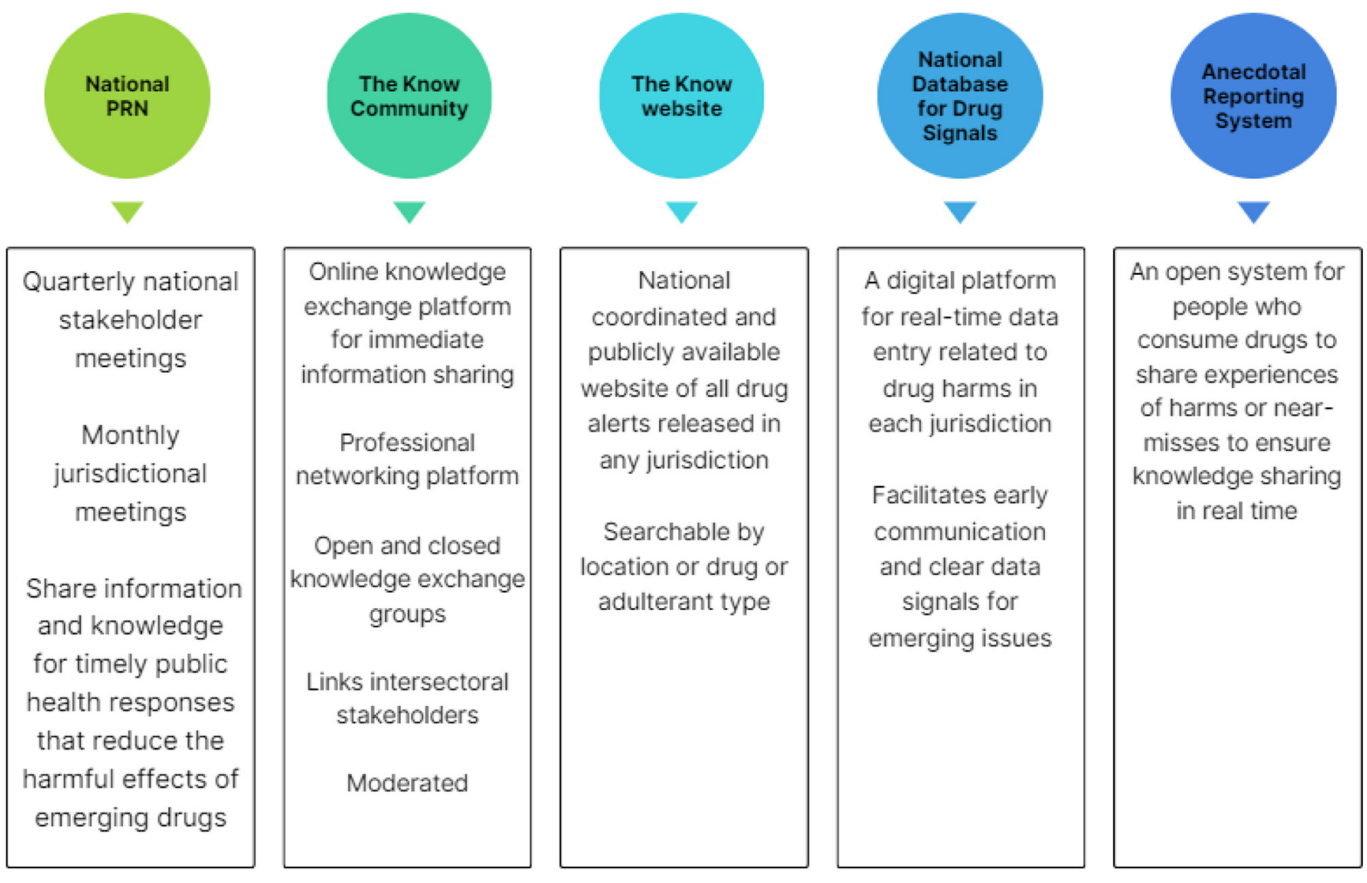
PRN components.

These platforms are innovative, offering cross-jurisdictional, and inter-sectoral collaboration. The PRN is coordinated by NCCRED to deliver these components, as shown in Figure 2.

## Outputs

*1. The National Prompt Response Network*

As a result of the co-design process, the PRN was formalized as a network engaging in information sharing. The National PRN is supported by a quarterly closed meeting that facilitates information exchange through updates from jurisdictional representatives and the national monitoring program Drug Trends (40) and the emerging emergency toxicosurveillance program EDNA (25). Additionally, these meetings provide capacity building through presentation by experts on subjects such as wastewater analysis or cryptomarket monitoring.

New multi-disciplinary stakeholders can be nominated to join by others in the group, and members are asked to nominate replacements if they change organizational roles. After the initial co-design phase was completed, the Jurisdictional Representative Group also chose to continue meeting monthly as a forum for discussing emerging drug signals and trends and sharing innovations in early warning system development. Each of these groups are supported by a closed online forum hosted in the online knowledge exchange community platform described below.

Following focus-group input from a selection of professional designs, a logo was selected to ensure consistent branding across outputs. Language guides and operating procedures were developed to guide the PRN through its growth.

*2. Online knowledge exchange platform “The Know Community*”

Participants in the co-creation process expressed the need to share information ahead of, and subsequent to, formal alerts (43), and to learn from the experiences of other jurisdictions. Collaborating across disciplines, obtaining anecdotal evidence, and gaining professional insights from peers or colleagues in other jurisdictions to access information earlier and ask questions to establish reach and scope of emerging risks, were seen as opportunities facilitated through the PRN.

Consultations identified challenges in recruiting key stakeholders from other states and territories working on similar projects, and in maintaining “key contact” lists when people change roles. This highlighted the need for networks that are role-dependent and systems-based, rather than relying on individuals and personal relationships. In response, NCCRED implemented a professional online network platform. Features are similar to other professional communities of practice, and designed with a structure similar to social media platforms, under the name “The Know Community”.

NCCRED conducted on-boarding for stakeholders involved with regional, jurisdictional, and national networks. This began in 2022 and continues on an opt-in basis or via reference from PRN members, with a broad and inclusive definition of people working within the field.

Within The Know Community, there is a live feed where members publish relevant posts on drug alerts, drug trend reports, or relevant articles. With NCCRED providing oversight and moderation, The Know Community serves as a national platform for collating resources, and offers registered members the opportunity to initiate closed conversations and confer via open or closed special-interest groups.

*3. National public drug alert website “http://theknow.org.au”*

State and territories issuing drug alerts publish them on their departmental websites, and local or regional organizations variously share these on their platforms. Before the development of “http://theknow.org.au”, a national approach to consolidating all alerts was not available, despite a growing need for a nationally cohesive picture [for example with emerging substances such as nitazenes reported variously across jurisdictions (7)]. Localized alerts limit consumers' ability to receive forewarning of information from other jurisdictions, and a national approach was seen as particularly valuable for communities close to interstate borders.

A co-design priority was to establish a public website to collate all drug alerts. In response, “http://theknow.org.au” was developed. Branding elements such as logos, imagery, and thematic schemes were co-designed through workshops with stakeholders from all reference groups. Focus groups were convened to consult on and select the final brand product “The Know”, ensuring its acceptability to people who use drugs.

“http://theknow.org.au” website was launched in 2023 and is maintained by NCCRED. It collates public health drug alerts issued nationally and provides relevant information in a curated and time-sensitive manner. Public drug alerts are searchable based on jurisdiction or drug type.

*4. National database for drug signals*

The fourth co-creation output in development is a national custom-built database for drug risk signals. This database is currently being designed to enable the systematic sharing of emerging drug-related concerns among Australian jurisdictions, supporting existing systems and facilitating a coordinated national approach. Data fields for this database were co-designed with jurisdictional representatives, including those from areas that currently lack formal infrastructure for collating signal data that could lead to public health warnings. This system addresses that gap and allows for flexibility given the diversity of signals and the varying data collections methods, with no mandatory data fields established. It is anticipated that data will be collated from multiple sources, and jurisdictional leads will enter data based on a perceived risk of harm, unexpected or novel phenomena, or cross-jurisdictional and national relevance. To ensure privacy, data will be cleaned upon entry to prevent potential re-identification and an administrator will manage data storage and user permissions.

A key challenge for this product is compliance with data security requirements, which have evolved and sometimes outpaced the co-design process. Additionally, agreements on data custodianship and sharing need to account for the legislative requirements of different jurisdictions, and work is ongoing to complete these.

*5. Anecdotal reporting system*

Initial scoping and consultation with the PRN Peer Advisory Group established the requirement for a moderated anecdotal reporting system. It was recognized that people who use drugs and service providers are often the first to notice emerging concerns. The anecdotal reporting system will provide people an opportunity to safely and anonymously report observations of new trends, harms or other concerns. It is envisaged that the coordinated collection of this information will complement other data sources through the broader PRN channels.

Furthermore, this system will support jurisdictions that have not yet implemented a system for issuing drug alerts, facilitating sharing of this information through the anecdotal system. Work continues on co-design with focus groups to further elucidate how the Anecdotal Information System might operate, where it will be accessed, and how it should be moderated.

## Discussion

The PRN brings together Australian agencies involved in responding to new and emerging drugs of concern to facilitate real-time information sharing, production of reliable resources, and opportunities for inter-disciplinary collaboration to address drug-related harms in Australia. Co-designed and co-produced with stakeholders nationally, the PRN connects people who use drugs, service providers, scientists, and key decision makers locally and nationally.

Although formal evaluation of the co-created products has yet to be finalized, there are learnings from the consultation, co-design, and co-production undertaken so far. The PRN demonstrates the sector's commitment to co-designing an integrated response to emerging drugs of concern in Australia. This is the first time in Australia that jurisdictions have a ready means for timely information sharing and a national approach to collect emerging signals. Effective engagement of policymakers, practitioners (from health and law enforcement) and consumer and peer organizations from the outset was key to the successful development and implementation of the PRN. Crucial to this was having a respectful understanding of the legitimate concerns and barriers that had prevented inter-jurisdictional and inter-sectoral sharing and cooperation, and affording time to build necessary trusting relationships between stakeholders. This also facilitated an understanding of how previous attempts at policy innovation had made stakeholders wary of top-down imposed “solutions,” overly academic approaches to data collection, and one-size-fits-all approaches.

Toxicological analysis is a central component of Australian drug information systems. The effectiveness of toxicology in informing public health responses to emerging concerns is contingent on sufficient toxicology capacity and the timely analysis of new substances. Effective drug monitoring for public health purposes needs to triangulate toxicological and anecdotal data from diverse sources in as timely a fashion as possible. This underscores the importance of maintaining a health-led approach and working with a wide range of stakeholders in co-designing the PRN.

The anecdotal reporting system will provide an opportunity for the PRN to work more closely with community information sources. People who use drugs are often the first to detect emerging drug trends, changes in supply, and new drug experiences. This system also presents a new paradigm for the PRN, whereby it will be information-generating in addition to networking and information sharing. To this end, implementing the broader frameworks and systems to support the anecdotal system was the initial step. Given the piecemeal availability of drug checking, there is extra imperative for anecdotal reporting systems to strengthen the knowledge held by people who use drugs and their experiences.

NCCRED first acted as a facilitator of co-design, and thereafter as a facilitator of the network during co-production. Having a separate Peer Advisory Group focused on the needs and capitalized on the knowledge of people who use drugs, and provided a “safe space” for their concerns to be elicited early in the co-creation process. This was also true for other stakeholder groups, making sufficient stakeholder mapping and analysis at the outset integral to the co-design and co-production steps.

There were a number of challenges to the co-creation process that merit noting. The COVID-19 pandemic occurred during the project. While this may have distracted the attention of many stakeholders and slowed down progress at certain points, it also increased the acceptability of working in online formats which simplified the process of cross-jurisdictional consultation and workshopping. Working with digital products with a large number of stakeholders with differing data security and operationalisation requirements requires expertise, and the co-design team eventually engaged its own digital product officer to manage the work of multiple IT projects and associated vendors. A unified structure for sharing sensitive data between the jurisdictions remains under development. Navigating data sharing that involves highly sensitive data and accounts for jurisdictional data protection requirements will necessarily take time to implement. Though jurisdictions vary in their needs, systems and resources, it is anticipated that once a data sharing system is in place, it can be built into emerging warning systems at the planning phase, supporting all regions to participate equally.

Though the final stage of co-creation is evaluation (39), this is intended to feed into a continuous process of iterative co-production. This relies on eliciting and responding to feedback from stakeholders so that the PRN has the flexibility to continue to evolve alongside local networks.

Through an evaluation of the PRN, several questions need to be considered alongside broader considerations around the continued facilitation of the network through an academic research center, the appropriate public health inputs and wider engagement that could be achieved and how best to enhance that capacity. The PRN has, as its core strength, a meaningful network of 88 member organizations nationally. Further resources remain under development, and thus the PRN has unrealised capacity. Current funding is allocated toward achieving the national database for drug signals, and for co-designing an anecdotal reporting system. However, in adapting to a fast-moving context, further resourcing will be required to bring these elements to full capacity. As is the case for most new public health initiatives, incremental achievements with a view to securing further resourcing once earlier phases have been met, is to be expected. A recurring feature of the organizations and individuals contributing to this project was that responding to NPS was only part of their role. While it is a limitation that the current funding is time-limited, it would be unrealistic to think that any public health program is not contingent on renewing funding, given the way that services are delivered and scaled in the context of public funding. As the PRN continues, and public health benefits are realized, the results achieved will be leveraged to lobby for ongoing support. Currently, jurisdictions have varied approaches to drug health policy and some have well developed systems of their own. While this heterogeneity is a limitation, the PRN is intended to mitigate this limitation by creating opportunities for intelligence of shared pertinence to be networked nationally. Rather than risk duplication of these, the PRN sees its role in complementing these through national collaboration, as well as providing capacity building and opportunity for triangulation of data sources for jurisdictions that do not have these resources. A unified early warning system should be a public health priority for the Australian government. The PRN is not designed to be binding, and its effectiveness thereby relies on active participation of stakeholders, including consumers. At this stage of network formation, consensus was an important mechanism to ensure that local needs were recognized and addressed while working at an inter-jurisdictional level. Future work may be enhanced by the development of legislative or regulatory frameworks to support participation, such as those which support communicable disease monitoring and control. However, careful thought as to how to encourage ongoing engagement to continue to improve the system is required.

Efforts have been made to establish such a coordinated response previously. The PRN's progress and engagement in this space is likely due to its co-design approach which respected the challenges and allowed time for relationships of trust to be developed. The PRN has a health focused response as its mission. Thus, the PRN was not framed as an early warning system, but as a facilitator of information sharing. The task of setting up the PRN as a national network in a federated country was a challenging but worthwhile endeavor in utilizing shared knowledge and co-creation of processes and platforms.

## Conclusions

Our learnings from the establishment and implementation of the PRN can benefit other localities/jurisdictions seeking to provide a similar shared network approach to emerging drug risks. The PRN continues to increase in relevance and value in the context of growing public health urgency related to an expanding NPS market. Key to establishing the PRN was its co-design and co-creation. Endeavors that engage meaningfully with co-design and co-creation require significant investment—mobilization of human and technology resources are essential. Providing a responsive network that is agile and does not replicate jurisdictional responses is imperative.

## Data Availability

The datasets presented in this article are not readily available because this community case study reports on a co-design of a public health program and does not include research data. Requests to access the datasets should be directed to krista.siefried@svha.org.au.
